# Evaluation of the relationship between stiffness and thickness of the sciatic nerve and clinical outcomes after total hip arthroplasty: A prospective case-controlled study

**DOI:** 10.5152/j.aott.2021.20324

**Published:** 2021-11-01

**Authors:** Osman Ciloğlu, Evren Karaali, Feride Fatma Görgülü, Emre Toğrul

**Affiliations:** 1 Department of Orthopaedic and Traumatology, University of Health Sciences Adana City Training and Research Hospital, Adana, Turkey; 2 Department of Radiology, University of Health Sciences Adana City Training and Research Hospital, Adana, Turkey; 3 Clinic of Orthopaedic and Traumatology, Ortopedia Hospital, Adana, Turkey

**Keywords:** Total hip arthroplasty, Leg lengthening, Sciatic nerve, Elastography

## Abstract

**Objective:**

The aim of this study was to conduct a sonographic assessment of sciatic nerve thickness and stiffness following total hip arthroplasty (THA) and to determine the relationship between sonographic characteristics of the sciatic nerve and clinical outcomes.

**Methods:**

This prospective study included patients undergoing primary cementless THA due to hip osteoarthritis between January 2018 and January 2019 in a tertiary-level hospital. The thickness, strain elastography, strain ratio (SR), and shear wave elastography (SWE) of the sciatic nerve were measured. The clinical outcome measures included leg lengthening (LL), leg length discrepancy (LLD), Oxford Hip Score (OHS), Visual Analog Scale (VAS) at rest, VAS during activity, and the Leeds Neuropathic Symptoms and Signs Evaluation (LANSS) scale. The data of the patient group were assessed preoperatively and at 3, 6, and 12 months postoperatively.

**Results:**

The sciatic thickness and SR values of the operated side were significantly lower than those of the non-operated side (*P* < 0.05 for all). The sciatic SWE was significantly greater on the operated side compared with the non-operated side (*P* < 0.001 for all). Sciatic nerve thickness and SR were negatively correlated, and sciatic nerve SWE was positively correlated with OHS, VAS at rest, VAS during activity, and LANSS values. Sciatic nerve thickness and SR values were significantly lower, and SWE was significantly higher in the group with a change in LL > 20 mm. Clinical scores decreased during the following period in mild and moderate lengthening group (< 20 mm). In the severe lengthening group (≥ 20 mm), the clinical score increased over time. The VAS activity score was higher in the mild and moderate LL group (< 20 mm) than the VAS rest score (*P* < 0.001). However, the VAS rest score was significantly higher in the severe LL group (≥ 20 mm) than the VAS activity score (*P* < 0.001).

**Conclusion:**

The results of this study have shown a significant relationship between thickness and stiffness of the sciatic nerve and LL after THA. The ultrasound parameters were significantly associated with functional outcomes.

**Level of Evidence:**

Level IV, Therapeutic Study

## Introduction

Total Hip Arthroplasty (THA) is a well-established procedure for severe hip Osteoarthritis (OA) patients, which reduces pain and enhances functionality.^1^ Obtaining an appropriate leg length without compromising the stability of the hip is one of the intraoperative challenges during THA.^2^ Leg Length Discrepancy (LLD) after THA most commonly involves over-lengthening, and Leg Lengthening (LL) of >1.5 cm has been reported to be poorly tolerated.^1,3^ Although the pathophysiology of the discomfort experienced by patients with LL is multifactorial, sciatic nerve pathologies have become a focus of research in recent years because of nerve traction resulting in LL following THA.^4^

Sciatic nerve abnormalities are also common following THA and may occur due to trauma during the surgical procedure, cement protrusion, trochanteric cable breakage, lateral displacement of the hip, or LL following the THA.^5,6^ Peripheral nerve Ultrasound (US) has been extensively employed in sciatic nerve imaging, as it has numerous advantages such as ease-of-application, low cost, repeatability, the absence of ionizing radiation, the provision of real-time imaging, and high spatial resolution.^7,8^ As a relatively new ultrasonographic technique, elastography provides valuable information about tissue stiffness.^9^ From a scan of literature, no study could be found, which has evaluated sciatic nerve stiffness and thickness using US following THA. Therefore, the objective of this study was to evaluate sciatic nerve thickness and stiffness using sonographic methods and to examine the correlations of these values with LL and clinical findings in patients following THA.

The study hypothesis was that stiffness would increase and thickness would decrease in the sciatic nerve due to LL following THA.

## Materials and Methods

### Study design and participants

**Figure 1.**
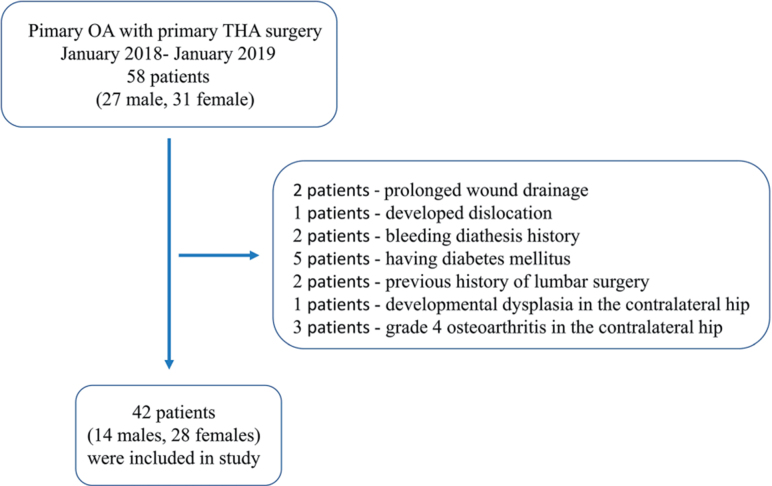
Flowchart for patient selection and exclusion criteria. OA, Osteoarthritis; THA: Total Hip Arthroplasty.

This prospective, case-controlled, and analytical study included patients who underwent primary cementless THA due to hip OA between January 2018 and January 2019 in a tertiary-level hospital. A total of 58 THA procedures were performed by a single surgeun (OÇ). Exclusion criteria included the presence of diabetes mellitus, polyneuropathy, a history of lumbar surger, sciatica, or bleeding diathesis, anatomic anomalies such as developmental dysplasia, coxa vara, grade 4 OA according to the Kellgren and Lawrence classification method,^10^ or collapse of the femoral head in the contralateral hip. In addition, patients applied with trochanteric cable-grip or plate, and patients who developed dislocation, or prolonged wound drainage in the follow-up period were also excluded from the study. Following implementation of the exclusion criteria, the study included a total of 42 patients who underwent THA, comprising 14 males and 28 females with a mean age of 55.93 ± 6.49 years and a mean Body Mass Index (BMI) of 24.07 ± 2.21 kg/m^2^ (Figure 1, Table 1).

All the surgical procedures were performed by a single surgeon. The patient was positioned lateral, and a posterolateral surgical approach was used as described by Moore.^11^ On postoperative day 1, patients were mobilized using crutches and were permitted weight-bearing as tolerated.

The radiological and ultrasonographic measurements of the operated and non-operated sides of each patient were recorded and compared. All the patients were assessed preoperatively and at 3, 6, and 12 months postoperatively.

Informed consent was obtained from all of the participants, and the study was conducted with the approval of the Institutional Review Board of our university hospital (No: 575/2019).

### Data collection and assessments

A record was made for each patient of clinical and demographic properties such as age, gender, BMI, type of anesthesia, comorbidities, and complications. The assessment measures were the Oxford Hip Score (OHS)^12^ and Visual Analog Scale (VAS) pain score (0–100) at rest and during activity.^13^ The OHS is a joint-specific questionnaire, which was developed and validated for use in THA patients, as a patient-reported assessment of functional ability and pain. The questionnaire comprises 12 items with 5-point Likert-type responses scored from 1 (no disability) to 5 (high disability), to give a final score in the range of 12–60. A higher score indicates a greater level of functional disability.^14^

The Turkish version of the Leeds Neuropathic Symptoms and Signs Evaluation (LANSS) Scale was also used. This is a useful tool for the differentiation of neuropathic patients from those with nociceptive pain, which is muscle, skeletal, and organ-based pain.^15^ The total score of the LANSS scale ranges from 0 to 24 with scores of ≥12 evaluated as neuropathic pain.

### Radiographic evaluation

**Figure 2.**
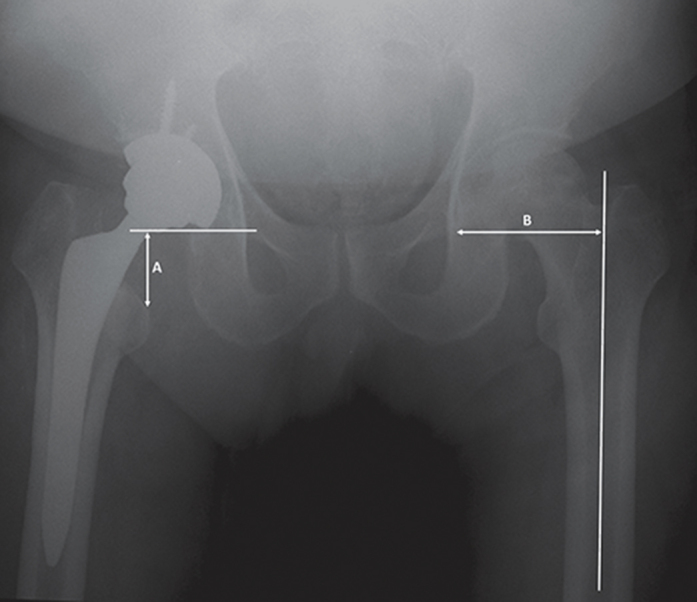
(A) Vertical offset; the distance between the line extending horizontally from the base of the teardrop and the medial apex of the trochanter minor. (B) Medial offset; the perpendicular distance from the teardrop base to the longitudinal axis of the femur.

Standard pelvis anteroposterior X-rays were taken preoperatively and postoperatively with the patient positioned supine with the legs in 15°–20° internal rotation and the knees in full extension. For the templating and measurements, the anatomic landmarks of ischial tuberosities, apex of the lesser trochanter, apex of the teardrop, and longitudinal axis of the femur were identified. The teardrop was used as the standard reference rather than the ischial tuberosity because previous studies have described the teardrop as a more consistent landmark.^16^ Vertical offset and medial offset measurements were taken using these anatomic landmarks (Figure 2). The vertical offset measured by the two observers was mean 69.42 and 64.27 mm, and the mean values for medial offset were 68.34 and 65.83 mm. Therefore, the measurement accuracy values were calculated as 91.4% and 94.6% with the precision of 0.77 and 0.37 mm, respectively. The acetabular cup diameter on the radiograph was measured and compared with that of the actual cup to assess the degree of magnification. Based on these measurements, differences in medial and vertical offsets (LLD) in the operated and non-operated limb were calculated after determining the differences in offsets from the preoperative and postoperative radiographs. A positive offset value was obtained when the involved side was longer or more lateralized than the contralateral side, whereas a negative value indicated the opposite. LL was measured as the difference between preoperative vertical offset and postoperative vertical offset on the operated side. Radiographic LLD was measured as the difference in the distance between the trans teardrop line and the most prominent point of the lesser trochanter in both hips.^17^ Patients were stratified into three subgroups based on LLD: 1) mild (0–10 mm); 2) moderate (10–19 mm); and 3) severe (≥20 mm).

### Sonographic evaluation

US examinations were performed by the same radiologist, experienced in musculoskeletal US and elastography, and blinded to the patient groups. The patients were evaluated using an US Doppler system (Philips EPIQ 7 Philips Health Care, Bothell, WA, USA) equipped with a linear high-resolution probe (5–18 MHz) (Philips L5-18). The sciatic nerve elastography from the gluteal region was performed using previously described.^8^ The patients were evaluated in the lateral decubitus position with the knees and hips in flexion.

The thickness of sciatic nerve in longitudinal plane was measured using the US Doppler machine electronic caliper. The thickness was accepted as the distance between the perineurium. The Strain Elastography (SE) images were graded automatically on the US device as follows: Grade 1: red to yellow (hardest or hard tissue), Grade 2: green (intermediate tissue), and Grade 3: blue (soft tissue).^18^

Shear Wave Elastography (SWE) measurements were obtained using a 1–5 MHz convex probe. Sciatic nerve stiffness was analyzed in Kilo Pascal (kPa) in the elasticity imaging range. Three measurements in a circular region of interest area were taken at 1–3 mm intervals. The Strain Ratio (SR), which is used to measure the stiffness of tissue, was automatically calculated by the US device, and the mean value of three measurements was used in the analysis.^19^

### Statistical analyses

The statistical analyses of the study were performed using the Statistical Package for Social Sciences (SPSS) 20.0 software (IBM SPSS Corp.;. Armonk, NY, USA). Descriptive statistics were presented as mean ± standard deviation (SD) and median values, and frequency and percentage. The conformity of continuous data to normal distribution was checked with the Shapiro–Wilk tests, and the variables were seen to have normal distribution (*P* > 0.05). The Independent Samples *t*-test was used for the comparisons of the study and control groups. Pre- and postoperative data were compared using the Paired Samples *t*-test. One-way ANOVA and Repeated Measures ANOVA were used to compare the clinical values between the LL groups and between preoperative follow-up months. Relationships between categorical variables were determined with the Chi-square test, and Pearson correlation analysis was performed to determine the relations between numerical variables. The multiple linear regression models were established to see the effects of clinical results on OHS, VAS, and VAS activity.

The reliability of the measurements was tested by two independent observers, and the operating surgeon was excluded from the measurement process to ensure objectivity. The observers performed radiographic measurements (medial offset and vertical offset) and were blinded to each other’s results. Intraclass Correlation Coefficient (ICC) analysis with the two-way mixed average measurement method was performed for agreement values. As the calculated values were between 0.918 and 0.983, the agreement for measurements was considered highly acceptable For SE, Kendall’s tau-b was used to determine the agreement between the observers, and the value was 0.877 (*P* < 0.001). The intra-observer measurements were evaluated by ICC with the one-way random consistency method. The results for the intra-observer measurements ranged between 0.932 and 0.993. A value of *P* < 0.05 was considered statistically significant with 5% type-I error.

## Results

The sciatic thickness and SR values of the operated side were significantly lower than those of the non-operated side (*P* < 0.05 for all). The sciatic SWE was significantly greater on the operated side compared to the non-operated side (*P* < 0.001 for all). In the SE evaluations, the hardest tissue (Grade 1: red to yellow) was the most common color code (59.5%) on the operated side of the patients, and intermediate tissue (Grade 2: green) was the most common code (61.9%) on the non-operated side (Table 2).

The preoperative and postoperative values are shown in Table 3. The vertical offset and vertical offset difference values were significantly different (*P* < 0.001), and there were no significant differences in terms of the medial offset and medial offset difference values between the preoperative and postoperative periods (*P* > 0.05).

**Figure 3.**
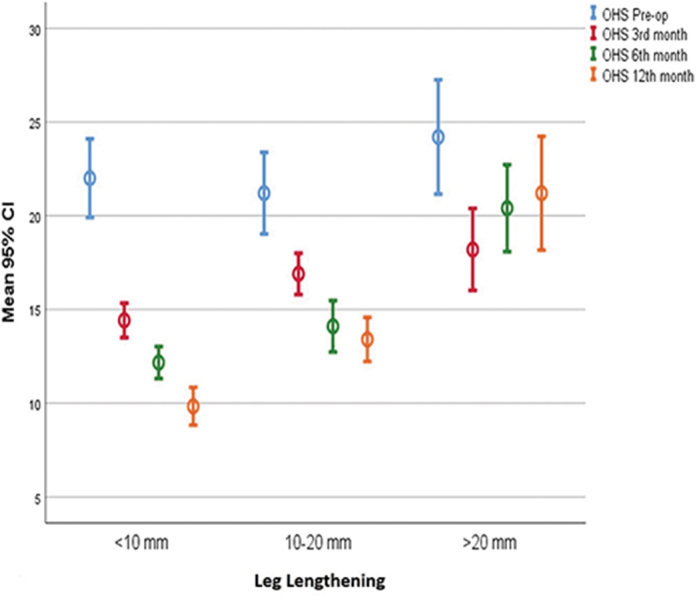
Changes in Oxford Hip Score over time according to the leg lengthening groups.

**Figure 4.**
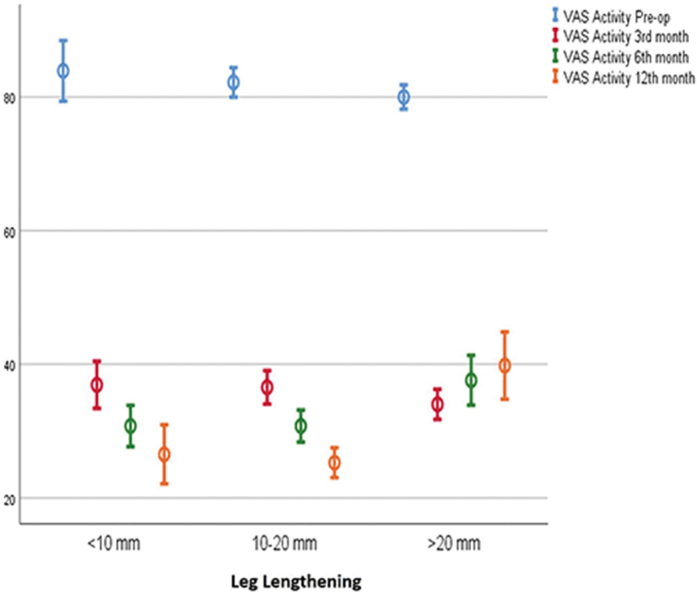
Changes in VAS activity scores over time according to the leg lengthening groups.

**Figure 5.**
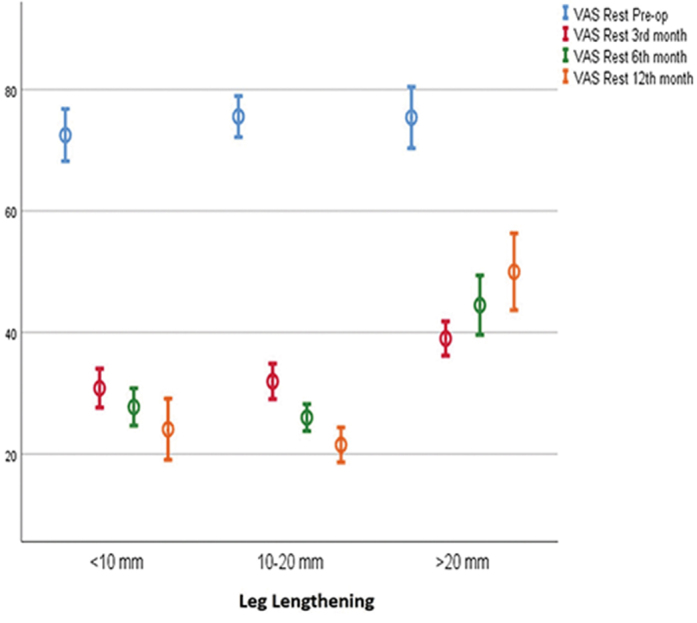
Changes in VAS rest scores over time according to the leg lengthening groups.

The US evaluations of the operated leg, according to the LL subgroups, are shown in Table 4. The sciatic nerve thickness and SR values were significantly lower, and the SWE was significantly higher in the severe lengthening group (≥20 mm) (*P* < 0.001 for all). There were no significant differences in terms of the medial offset and medial offset difference values between the LL subgroups (*P* > 0.05). The clinical scores decreased during the following period in the mild and moderate lengthening groups (<20 mm). In the severe lengthening group (≥20 mm), the clinical score increased over time (Figure 3**–**5).

**Figure 6.**
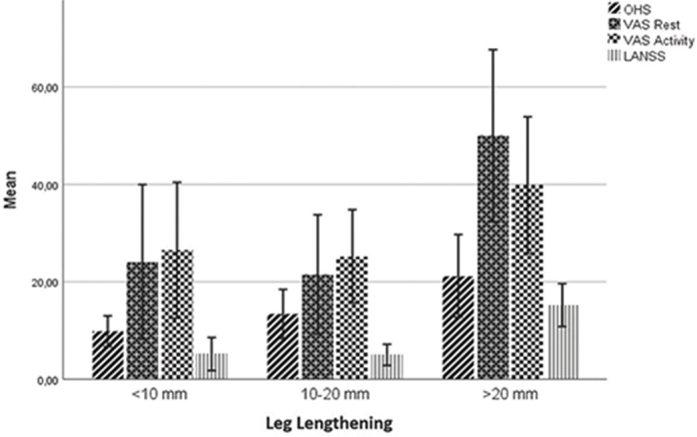
Clinical outcomes at 12 months postoperatively, according to the leg lengthening groups.

The VAS activity score was higher than the VAS rest score in the mild and moderate LL groups (<20 mm) (*P* < 0.001). In the severe LL group (≥20 mm), the VAS rest score was significantly higher than the VAS activity score (*P* < 0.001) (Figure 6).

The correlation analysis results are shown in Table 5. LL showed a strong significant correlation (*P* < 0.001) with OHS (*r* = 0.851), VAS rest (*r* = 0.730), LANSS scores (*r* = 0.834), and a moderate correlation with VAS activity (*r* = 0.617). LLD was not significantly correlated with the clinical scores (*P* > 0.05).^20^

The regression analysis results, showing the effects of parameters on clinical results, are shown in Table 6. The contribution of US values and LL were significant to the model (*P* < 0.05 for all), especially in the severe LL group (≥20 mm) (*P* < 0.001). No significant effect of LLD on clinical results was determined (*P* > 0.05).

### Discussion

The current study had three main outcomes. First, the sciatic nerve thickening, stiffness, and elasticity values changed following THA. Second, the US parameters were significantly correlated with the functional outcomes, and finally, LL showed a significant correlation with the clinical scores, although LLD was not correlated.

Many etiological factors could contribute to sciatic nerve abnormalities following THA. However, significant emphasis has been placed on LL as one possible factor.^17,21^ Other factors that have been reported include 1) lateral displacement of the hip^22^; 2) direct retraction or trauma to the nerve^23^; and 3) damage by trochanteric reattachment.^24^ However, to the best of our knowledge, the relationship between LL and the sciatic nerve following THA, and the effect on clinical functions, has not been previously investigated using US. In this study, the effect of LL on the sciatic nerve in relation to the clinical scores was evaluated by excluding patients who had been applied with a cemented stem, trochanteric plate, cable, or wire, as these could affect the sciatic nerve injury. Furthermore, no significant differences were observed between the patients when the medial offset values of the contralateral side were compared. Therefore, the study can be considered of value in respect of the comparison of the medialization–lateralization of the hip between groups using similar parameters as the LL and sciatic nerve sonographic data.

US has previously been used to evaluate the sciatic nerve and is a valid and reliable method.^7,8,25^ Elastography is a relatively new imaging technique that is used to quantiatively evaluate tissue elasticity and stiffness. The two main elastographic methods are SE and SWE. SE is semiquantitative and more user-dependent, considering that the sonographer applies the compression, whereas SWE provides quantitative and direct characteristics of elasticity as the transducer applies the compression.^26-28^ Therefore, both of these elastographic methods were used in the current study to achieve more reliable results. There is a limited number of studies in the literature regarding sciatic nerve elastography. In the present study, the first notable finding was the significant change observed in sciatic nerve stiffness in the involved side compared with that in the non-operated side of the patients.

Sciatic nerve thickness and SR were decreased, and SWE was increased in the mild and moderate LL subgroups (0–10 and 10–20 mm). This can be attributed to the traction and stress on the sciatic nerve. In the follow-up group, the sciatic nerve US values returned to the level of those on the non-operated side. In contrast, LL was more than 20 mm (severe lengthening) in the early postoperative third month, and US values were similar within the mild and moderate LL groups, although the values did not return to the same level as recorded for the non-operated leg. The sciatic thickness and SR were significantly lower, and SWE was significantly higher in the 12-month follow-up examination. Persistent inflammation due to the duration of the symptoms could result in demyelination and changes in the connective tissue of the nerve, such as atrophy, and fibrosis. The correlation analyses showed that it is less tolerated by the patients.

The clinical scores were significantly positively correlated with the US values of the sciatic nerve (*P* < 0.001 for all) (Table 4). The VAS rest score was significantly higher than the VAS activity score only in patients with LL ≥ 20 mm (Figure 4). In this context, although this group of patients might have higher pain at rest, it could be due to the sciatic nerve rather than the osseous or endoprosthesis; therefore, this result should be supported by Electromyography (EMG). The evaluation using the LANSS pain scale also showed that the rate of neuropathic pain was higher in the severe lengthening group (≥20 mm) after THA.

The boundary between acceptable and unacceptable discrepancies has not yet been clarified. Previous studies have reported that patients could clinically tolerate LLD of up to 10 mm, which may go unnoticed, LLD of 10–20 mm can be successfully treated with physical therapy, although the functional outcome scores can be negatively affected, whereas higher LLD values can lead to patient dissatisfaction, nerve palsy, and back pain.^29-31^ Lengthening of the contralateral limb has been stated to be a treatment option that is effective for LLD following THA when no other non-surgical treatment options are successful.^31,32^ However, if this dissatisfaction is due to sciatic nerve elongation, it can be thought that contralateral limb lengthening surgery would be ineffective. Therefore, determination of the etiology is vital in the management of dissatisfaction. There is currently no algorithm for the management of symptomatic limb length discrepancies after THA.^32^

In the development of LLD after THA, clinical dissatisfaction is anticipated. As patients may have preoperative leg shortness due to arthritic changes in the involved joint, postoperative LL may occur without LLD after THA. It may sometimes be difficult for surgeons to determine the etiology of pain in such patients. In the current study, it was thought that LL as well as LLD may assist in determining the etiology of pain. Although the results of this study did not show a correlation of LLD with the clinical results, LL was seen to be significantly correlated, which supports this hypothesis. In this context, after excluding other factors such as infection, stem loosening, and malpositioning, which may cause pain and clinical complaints, US assessment can be considered to assist in the evaluation of sciatic nerve morphology, especially if LL is present, with/without significant LLD.

EMG is acknowledged as the gold standard in the assessment and diagnosis of nerve injury.^33^ However, US has some advantages as it is a non-invasive, reproducible, easy-to-apply method, and provides a morphological view prior to histopathological changes occurring in the nerve.^34^ Therefore, it can be considered to contribute to patient follow-up after THA with LL. The regression analysis results are shown in Table 5.

This study had some limitations. The lack of electrodiagnostic testing and direct comparison between US parameters and electrodiagnostic parameters can be said to be a limitation of the study, and that the cross-sectional area of the nerve could have been measured rather than the thickness. The patient position during US measurement process could constitute another limitation. The main positon to relax the sciatic nerve is extension of the hip and flexion of the knee. In this study, patients were positioned with both the hips and knees in flexion as this position has been used in several previous studies, although the sciatic nerve in this position would be stiffer than in the relaxed positon. The US measurements were taken by a single observer, so the lack of interobserver reliability of the measurements could be said to be another limitation. The lack of a control group is a further limitation.

In this study, the clinical scores of LL up to 20 mm decreased over time following THA. However, a significant change in the sciatic nerve thickness and stiffness was seen in patients with a lengthening of ≥20 mm after THA because it was found to be associated with worse outcomes that failed to regress over time. Therefore, surgeons need to keep in mind that patients with LL of >20 mm and clinical symptoms should receive further investigation and treatment.

In the light of these results, the association between sciatic nerve thickness and stiffness has been shown with LL after THA. USG may be a supportive guide in LL management following THA. As this is the first study on this subject, there is a need for further studies with larger sample sizes and long-term follow-up periods to be able to confirm these findings.

**Highlights**

- This study aimed to evaluate the sciatic nerve thickness and stiffness following a Total Hip Arthroplasty (THA) using sonographic methods.
- The sciatic thickness and strain ratio values of the operated side were significantly lower than those of the non-operated side
- Sciatic nerve thickness and strain ratio values were significantly lower in the group with a change in Leg Lengthening (LL) greater than 20 mm.
- The ultrasound parameters are also significantly associated with functional outcomes.
- Ultrasonography can successfully be used to examine sciatic nerve morphology in daily clinical practice, and it should be a supportive guide in LL management following THA.

## Figures and Tables

**Table 1.** Patient Characteristics

Characteristics	Total Patients (*n* = 42)	Mild Group (LL < 10 mm) (*n* = 12)	Moderate Group (LL: 10–19 mm) (*n* = 20)	Severe Group (LL: ≥20 mm) (*n* = 10)	
	Mean ± SD				*P*
Age (years)	55.93 ± 6.49	58.42 ± 7.37	57.15 ± 5.44	50.5 ± 4.33^+^	*0.006* *
BMI (kg/m^2^)	24.07 ± 2.21	24.25 ± 2.05	24.45 ± 2.56	23.1 ± 1.37	*0.280*
	*n* (%)				*P*
Gender					
Male	14 (33.3)	1 (8.3)	7 (35.0)	6 (60.0)	*0.011* **
Female	28 (66.7)	11 (91.7)	13 (65.0)	4 (40.0)	

* significant at *P* < 0.05 level according to one-way ANOVA.

+ significantly different pairwise group according to Tukey HSD test.

** significant at *P* < 0.05 level according to Chi-square test.

**Table 2.** Comparison of Radiological and Ultrasonographic Values Between the Operated Leg and the Non-Operated Leg

	Patients		
	Operated Leg (*n* = 42)	Non-Operated Leg (*n* = 42)	
	Mean ± SD		*P*
Vertical offset (mm)	67.61 ± 4.31	59.78 ± 6.47	*<0.001* *
Medial offset (mm)	66.85 ± 3.83	72.90 ± 4.24	*<0.001* *
Sciatic thickness (mm)***	5.30 ± 0.63	5.64 ± 0.21	*<0.001* *
Sciatic strain ratio***	7.74 ± 1.52	8.54 ± 0.21	*0.002* *
Sciatic shear wave elastography (kPA)***	6.97 ± 2.69	5.34 ± 0.19	*<0.001* *
Strain elastography, *n* (%)*** - Red-yellow (hard) - Green - Blue (soft)	25 (59.5) 13 (31.0) 4 (9.5)	7 (16.7) 26 (61.9) 9 (24.1)	*<0.001* **

* significant at *P* < 0.05 level according to Student’s *t*-test.

** significant at *P* < 0.05 level according to Chi-square test.

*** 12-month follow-up measurement.

**Table 3.** Comparison of Preoperative and Postoperative Radiological and Clinical Scores on the Operated Side

Operated Leg (*n* = 42)	Preoperative	Postoperative	
	Mean ± SD		*P*
Vertical offset (mm) Vertical offset diffrence (mm)**	53.21 ± 112.23 −6.57 ± 10.72	67.61 ± 4.31 +7.83 ± 4.35	*<0.001* * *<0.001* *
Medial offset (mm) Medial offset difference (mm)**	70.35 ± 3.92 −4.38 ± 1.76	69.88 ± 3.94 −4.59 ± 1.56	*0.141* *0.108*
Oxford Hip Score*** Visual Analog Scale rest*** Visual Analog Scale activity***	22.14 ± 4.29 74.64 ± 7.01 82.16 ± 5.24	14.23 ± 5.04 29.02 ± 13.90 29.07 ± 5.24	*<0.001* * *<0.001* * *<0.001* *

* significant at *P* < 0.05 level according to Paired Sample *t*-test.

** the difference between operated and non-operated side.

*** 12-month follow-up measurement.

**Table 4.** Comparison of the Radiological and Ultrasonographic Measurements of the Operated Leg According to Leg Lengthening Groups

Operated Leg (*n* = 42)		Mild Group (LL < 10 mm) (*n* = 12)	Moderate Group (LL: 10–19 mm) (*n* = 20)	Severe Group (LL: ≥ 20 mm) (*n* = 10)	*Between Groups*
	Postoperative	Mean ± SD			*P*
Sciatic thickness (mm)					
	*3^rd^ month*	5.77 ± 0.35	5.33 ± 0.17	4.69 ± 0.42	*<0.001* *
	*6^th^ month*	5.70 ± 0.27	5.44 ± 0.16	4.55 ± 0.44	*<0.001* *
	*12^th^ month*	5.70 ± 0.19	5.55 ± 0.18	4.31 ± 0.52	*<0.001* *
	*Within groups P*	*<0.001* **			
Strain ratio					
	*3^rd^ month*	8.75 ± 0.73	7.18 ± 0.53	5.42 ± 0.58	<0.001*
	*6^th^ month*	8.80 ± 0.21	7.97 ± 1.35	5.33 ± 0.62	*0.001* *
	*12^th^ month*	8.69 ± 0.14	8.51 ± 0.14	5.09 ± 0.65	<0.001*
	*Within groups P*	*<0.001* **			
Shear wave elastography (kPA)					
	*3^rd^ month*	5.59 ± 0.82	7.24 ± 0.81	10.46 ± 2.18	*<0.001* *
	*6^th^ month*	5.56 ± 0.21	6.46 ± 0.58	10.85 ± 0.26	*<0.001* *
	*12^th^ month*	5.70 ± 0.17	5.69 ± 0.20	11.06 ± 2.90	*<0.001* *
	*Within groups P*	* <0.001* **			
Vertical offset (mm)		68.41 ± 3.42	69.50 ± 2.68	62.90 ± 4.67	* <0.001* *
Vertical offset difference (LLD) (mm)***		8.08 ± 5.07	8.25 ± 3.27	6.70 ± 5.49	*0.648*
Medial offset (mm)		69.00 ± 2.52	70.15 ± 4.42	70.40 ± 4.50	*0.660*
Medial offset difference (mm)***		−4.08 ± 1.88	−4.70 ± 1.30	−5.00 ± 1.63	*0.368*

* significant at *P* < 0.05 level according to one-way ANOVA.

** significant at *P* < 0.05 level according to Repeated Measure ANOVA.

*** the difference between operated and non-operated side.

LLD, leg length discrepancy.

**Table 5.** Correlation Analysis Results of the Radiological and Ultrasonographic Measurements and Clinical Scores

Variables		OHS	VAS Rest	VAS Activity	LANSS
Leg length discrepancy (mm)	*r*	−0.112	−0.041	−0.065	−0.119
	*P*	*0.482*	*0.794*	*0.684*	*0.451*
Leg lengthening (mm)	*r*	0.851	0.730	0.617	0.834
	*P*	*<0.001* *	*<0.001* *	*<0.001* *	*<0.001* *
Sciatic thickness (mm)**	*r*	−0.789	−0.808	−0.675	−0.900
	*P*	*<0.001* *	*<0.001* *	*<0.001* *	*<0.001* *
Sciatic strain ratio**	*r*	−0.828	−0.872	−0.735	−0.949
	*P*	*<0.001* *	*<0.001* *	*<0.001* *	*<0.001* *
Sciatic SWE (kPA)**	*r*	0.738	0.821	0.673	0.871
	*P*	*<0.001* *	*<0.001* *	*<0.001* *	*<0.001* *

* correlation is significant at the 0.01 level according to Pearson Correlation Analysis.

** 12-month follow-up measurement.

OHS, Oxford Hip Score; VAS, Visual Analog Scale; SWE, Shear Wave Elastography; LANSS, Leeds Neuropathic Symptoms and Signs Evaluation Scale.

**Table 6.** Multiple Linear Regression Analysis of Parameters of Clinical Results

Clinical Findings	Variables	Unstandardized Beta	Standardized Beta	*P*
OHS				
	*Thickness* (mm)	−6.253	0.789	*0.001* *
	*Strain ratio*	−2.706	−0.828	*0.011* *
	*SWE* (kPA)	1.383	0.738	*0.017* *
	*LLD* (mm)	−0.129	−0.112	*0.482*
	*LL* (mm)	0.460	0.851	*0.008* *
	*LL (<20 mm)*	1.123	0.215	*0.089*
	*LL (≥20 mm)*	9.138	0.781	*<0.001* *
VAS rest				
	*Thickness* (mm)	−17.651	−0.808	*0.003* *
	*Strain ratio*	−7.861	−0.872	*0.002* *
	*SWE* (kPA)	4.424	0.821	*0.004* *
	*LLD* (mm)	−0.133	−0.041	*0.794*
	*LL* (mm)	1.088	0.730	*0.018* *
	*LL (<20 mm)*	1.614	0.513	*0.138*
	*LL (≥20 mm)*	27.531	0.853	*<0.001* *
VAS activity				
	*Thickness* (mm)	−8.970	−0.675	*0.002* *
	*Strain ratio*	−4.029	−0.735	*0.005* *
	*SWE* (kPA)	2.117	0.673	*0.016* *
	*LLD* (mm)	−0.126	−0.065	*0.684*
	*LL* (mm)	0.559	0.617	*0.028* *
	*LL (<20 mm)*	1.267	0.548	*0.106*
	*LL (≥20 mm)*	14.081	0.718	*<0.001* *

* significant at *P* < 0.05 level according to multiple linear regression.

OHS, Oxford Hip Score; VAS, Visual Analog Scale; SWE, Shear Wave Elastography; LLD, Leg Length Discrepancy; LL, Leg Lengthening.
